# Silica Desiccant Packets for Storage and Transport of *Streptococcus pneumoniae* and Other Clinically Relevant Species

**DOI:** 10.1371/journal.pone.0072353

**Published:** 2013-08-07

**Authors:** Casey L. Pell, Melanie J. Williams, Eileen M. Dunne, Barbara D. Porter, Catherine Satzke

**Affiliations:** 1 Pneumococcal Research, Murdoch Childrens Research Institute, Royal Children’s Hospital, Parkville, Victoria, Australia; 2 Department of Microbiology and Immunology, the University of Melbourne, Parkville, Victoria, Australia; Centers for Disease Control & Prevention, United States of America

## Abstract

Bacterial isolates are often transported between laboratories for research and diagnostic purposes. Silica desiccant packets (SDPs), which are inexpensive and do not require freezing, were evaluated for storage and recovery of bacterial isolates. Conditions such as inoculum size, swab type and temperature of storage were investigated using ten *Streptococcus pneumoniae* isolates. The optimized protocol was then tested using 49 additional *S. pneumoniae* isolates representing 40 serogroups. Overall, *S. pneumoniae* growth was considered satisfactory (>100 colony forming units) for 98/109 (89.9%) and 20/20 (100%) swabs after 14 days at room temperature or 28 days at 4° C, respectively. Storage in SDPs did not impact on the ability of *S. pneumoniae* isolates to be subsequently serotyped. When the survival of nine other clinically relevant bacterial species was tested, seven were viable after 28 days at room temperature, the exceptions being *Neisseria gonorrhoeae* and *Haemophilus influenzae*. SDPs are suitable for transport and short-term storage of bacterial species including *S. pneumoniae*.

## Introduction

*Streptococcus pneumoniae* is an important pathogen, particularly in low-income countries [1]. *S. pneumoniae* isolates are often transported internationally during clinical or research studies. *S. pneumoniae* isolates can be transported in skim milk, tryptone, glucose, glycerol (STGG) medium, on agar slants, or following lyophilization [2–4]; and Stuart’s, Aimes’ or STGG transport media are commonly used for nasopharyngeal swab transport [2,4]. Selective egg-based media is effective for storage of nasopharyngeal swabs at ambient temperatures up to seven days [4]. Silica desiccant packets (SDPs), an approach similar to sand desiccation [3], have been used to transport bacterial species including *Corynebacterium diphtheriae, Staphylococcus aureus, Streptococcus pyogenes*, and *Neisseria* *meningitidis* [5–9]. SDPs were also used for transporting nasopharyngeal swabs likely to contain *S. pneumoniae* [10], a species surprisingly tolerant of desiccation [11]. SDPs are a simple and economical transport method. The Centers for Disease Control and Prevention suggests that SDPs are suitable for short-term storage of *S. pneumoniae* isolates [12], however, optimal inoculation and storage conditions remain undefined. This study investigated the impact of inoculum size, swab type and storage temperature on the recovery of ten *S. pneumoniae* isolates from SDPs. The effects of SDP storage on colony morphology and the ability to be serotyped were also evaluated. The optimized protocol was then further tested with 49 additional *S. pneumoniae* isolates and nine other clinically relevant bacterial species.

## Materials and Methods

### Bacterial isolates

Ten *S. pneumoniae* isolates: PMP435 (serotype 6A), PMP492 (serotype 6B), PMP484 (serotype 6B), PMP814 (serotype 8), PMP288 (serotype 10A), PMP815 (serotype 12F), PMP122 (serotype 13), PMP63 (serotype 19F), PMP713 (serotype 23F), PMP821 (serotype 31) were initially examined, followed by an additional 49 *S. pneumoniae* isolates representing 40 serogroups (Table S1). Clinical isolates of *Escherichia coli, Haemophilus influenzae, Klebsiella pneumoniae, Neisseria gonorrhoeae, Pseudomonas aeruginosa*, S*almonella spp., Staphylococcus aureus, Streptococcus agalactiae* and *Streptococcus pyogenes* (Table S1) were kindly provided by The Department of Microbiology, Laboratory Services, Royal Children’s Hospital, Parkville, Australia. Isolates were recovered from storage in STGG or Microbank Protect beads at -80^°^ C prior to use. Isolates were plated on horse blood agar (HBA) plates (Oxoid brand, Thermo, Fisher Scientific, Australia) as lawn cultures, and incubated for 18-24 h at 37^°^ C, 5% CO_2_. Chocolate agar plates (Oxoid brand, Thermo, Fisher Scientific, Australia) were used for *N. gonorrhoeae* and *H. influenzae* strains, with an additional 24 h incubation given at each culture step for *N. gonorrhoeae*. These plating and incubation conditions were used throughout the study.

### SDP inoculation, storage and recovery

i The core methodology used is outlined here. In duplicate, polyester-tipped (Copan Diagnostics) or nylon-flocked (Copan Flock Technologies) swabs were used to harvest one-quarter lawn of growth, then placed into separate SDPs (Sud-Chemie, Australia), sealed and stored at room temperature for up to 28 days. This storage process was repeated for each time point tested. On recovery, swabs were removed from SDPs and plated for lawn growth. Several drops of sterile Todd-Hewitt broth (THB) were applied to the swab until the entire tip was moistened, using a sterile transfer pipette, prior to plating. After incubation, colony morphology was examined. Growth was assessed by viable count and categorized as TNTC (too numerous to count; >1000 CFU), heavy (100-1000 CFU), moderate (20-99 CFU), light (<20 CFU) or no growth; adapted from Popovic et al. [8]. Growth was further defined as satisfactory (≥100 CFU) or unsatisfactory (<100 CFU) based on the number of colonies required for techniques such as serotyping from the primary recovery plate. Statistical analyses were performed with Fisher’s exact test; *P* <0.05 was considered significant.
ii At times, the core methodology was varied. This is indicated in the text, but in brief: to investigate the effect of swab type, cotton (Handy, BDF Australia) and Rayón-tipped (Copan Diagnostics) swabs were compared with polyester-tipped and nylon-flocked by inoculating four swabs for each isolate. Growth was assessed after 7 and 14 days, with one swab of each pair plated directly instead of being moistened with THB. To investigate the effect of inoculum size, one-half or full lawn cultures were also used. Ten swabs were inoculated per isolate for each inoculum and recovery assessed after 4, 7, 10 14 and 28 days. To investigate the effects of temperature, duplicate swabs were inoculated and one of each pair stored at 4° C for 7, 14 or 28 days. To investigate the effects of moistening, one swab of each pair was plated directly, instead of being moistened with THB. All variations were performed on a single occasion and analyzed as for the core method, except that the Mann-Whitney test was used for the effect of swab type comparisons (with *P* <0.05 considered significant).

### Identification and serotyping

Before storage and after recovery, *S. pneumoniae* isolates were serotyped using a combination of commercially available reagents (Denka Seiken Co. Ltd, Japan) and latex agglutination reagents prepared in-house from Statens Serum Institut antisera [13]. Clinical isolates were identified with VITEK^®^ (bioMérieux). Serological tests were also performed for *S. agalactiae* and *S. pyogenes* (PROLEX™ Streptococcal grouping latex kit, Pro-Lab Diagnostics)*, Salmonella spp.* (Wellcolex* Colour Salmonella, Remel) and *S. aureus* (PROLEX™ Staphylococcus Xtra Latex kit, Pro-Lab Diagnostics)*.*

## Results and Discussion

Firstly, the impact of swab type on recovery of ten *S. pneumoniae* isolates was compared. After 7 days, all swabs produced growth TNTC, with the exception of 3 of 20 (15%) Rayón and 1 of 20 (5%) cotton swabs that had heavy growth. At 14 days, a total of 77 of 80 (96%) swabs yielded growth, with growth TNTC for 7/20 (35%), 9/20 (45%), 11/20 (55%), and 12/20 (60%) swabs for Rayón, cotton, polyester and nylon-flocked swabs, respectively. There were no significant differences between swab types in the percentage yielding TNTC growth at 7 and 14 days (data combined) (*P* >0.05). However, analysis of total CFU counts using Mann Whitney test for the dry swabs showed Rayón (132.4 (95% CI: -87.1, 352.9)) to be inferior to both polyester (625.1 (95% CI: 324.2, 926.0)) and nylon-flocked swabs (562.9 (95% CI: 225.6, 899.6)), *P* =0.01 and *P* =0.03, respectively. There was no significant difference between comparisons with cotton swabs (382.7 (95% CI: 66.24, 699.2)) or when moistened swabs were analyzed separately (see Materials and Methods, data not shown). Subsequently, polyester and nylon-flocked swabs were used for all following experiments.

Effects of inoculum size on recovery were investigated by inoculating swabs with quarter, half or full lawn cultures. Growth was assessed after 4, 7, 10, 14 and 28 days of storage. Growth was recoverable from all swabs, irrespective of inoculation size, after 4, 7 and 10 days; with 94 of 100 (94%) swabs recovering heavy growth after 14 days. At 28 days, all inocula showed reduced recovery of satisfactory growth (data not shown), and half-lawns yielded more recovery of satisfactory growth compared with quarter-lawns (*P* =0.0489). No other significant differences were found between inocula (*P* >0.05). As 14 days was deemed an acceptable time limit for transport, one-quarter lawns were chosen for subsequent experiments, as this smaller amount of inoculum was sufficient to recover satisfactory growth following SDP storage for the isolates tested.

Moistening swabs with THB prior to plating was then further evaluated. On recovery, swabs were removed from SDPs with one plated directly, the other moistened first with THB. After 14 days (Table 1), a combined total of 39 of 40 (98%) swabs produced growth, with both moistened and dry swabs each yielding satisfactory growth in 15 of 20 (75%) swabs. The moistened method resulted in 13 of 20 (65%) swabs with growth TNTC, compared with 10 of 20 (50%) for the dry method. Although no significant differences were seen (*P* >0.05), the moistened method was chosen for subsequent experiments as the moistened swabs caused less damage to the agar surface.

**Table 1:** S. pneumoniae growth recovered from swabs stored in SDPs at room temperature and plated dry or after moistening.

		No. (%) swabs^*a*^			
		Dry method		Moistened method	
Category^*b*^	Growth^*c*^	Day 7	Day 14	Day 7	Day 14
Satisfactory	TNTC	20 (100)	10 (50)	20 (100)	13 (65)
	Heavy	0 (0)	5 (25)	0 (0)	2 (10)
Unsatisfactory	Moderate	0 (0)	1 (5)	0 (0)	2 (10)
	Light	0 (0)	4 (20)	0 (0)	2 (10)
	No growth	0 (0)	0 (0)	0 (0)	1 (5)
	Total	20 (100)	20 (100)	20 (100)	20(100)

^a^ Data from single experiment of n=10 polyester-tipped, n=10 nylon-flocked swabs. No significant difference between swabs types (P >0.05) ^b^ Satisfactory growth, ≥ 100 CFU/plate; unsatisfactory growth, <100 CFU/plate. ^c^ TNTC, >1000 CFU; Heavy, 100-1000 CFU; Moderate, 20-99 CFU; Light, <20 CFU; and No growth, 0 CFU.

To examine the effect of temperature, swabs were stored in SDPs at room temperature or 4^°^ C for up to 28 days. At room temperature (Table 2) all swabs produced growth TNTC after 7 days, with the exception of one polyester swab which was lost to follow up. Following 14 days storage, 59 of 60 (98%) swabs produced growth with 54 (90%) swabs recovering satisfactory growth. When storage times were extended to 28 days, recovery decreased significantly (*P* =0.0001); 32 of 40 (80%) swabs recovered growth with only 12 (30%) swabs yielding satisfactory growth. Reduced growth was not observed over the 28 days when swabs were stored at 4^°^ C, with all swabs (n =60) yielding growth TNTC. At 14 days, results for the two storage temperatures were comparable. Recovery after 28 days was significantly better (*P* =0.0001) when swabs were stored at 4^°^ C. These results suggest that in most cases, 14 days is a suitable duration for storage in SDPs at room temperature, and storage can be extended to 28 days at 4^°^ C. This extends the storage limits previously published [12]. Further investigations could assess suitability of longer storage times at 4^°^ C.

**Table 2:** *S. pneumoniae* growth recovered from swabs stored in SDPs at room temperature and 4˚C.

		No. (%) swabs^*a*^					
		Room temperature			4˚C		
Category^*b*^	Growth ^*c*^	Day 7	Day 14	Day 28	Day 7	Day 14	Day 28
Satisfactory	TNTC	59^*d*^ (100)	47 (78)	8 (20)	20 (100)	20 (100)	20 (100)
	Heavy	0 (0)	7 (12)	4 (10)	0 (0)	0 (0)	0 (0)
Unsatisfactory	Moderate	0 (0)	2 (3)	9 (23)	0 (0)	0 (0)	0 (0)
	Light	0 (0)	3 (5)	11 (28)	0 (0)	0 (0)	0 (0)
	No growth	0 (0)	1 (2)	8 (20)	0 (0)	0 (0)	0 (0)
	Total	59 (100)	60 (100)	40 (100)	20 (100)	20 (100)	20 (100)

^*a*^ **Data represents three (room temperature Day 7 and 14), two (room temperature Day 28) and one (4˚C all time points) independent experiments of n=10 polyester-tipped and n=10 nylon-flocked swabs.

^*b*^ Satisfactory growth, ≥ 100 CFU; unsatisfactory growth, <100 CFU.

^*c*^ TNTC, >1000 CFU; Heavy, 100-1000 CFU; Moderate, 20-99 CFU; Light, <20 CFU; and No growth, 0 CFU.

^*d*^ Sixty swabs were inoculated, however one polyester-tipped swab was lost to follow up

Unfavorable storage conditions may influence colony morphology and the ability to serotype *S. pneumoniae* isolates [14]. We examined effects of SDP storage on these phenotypes by observing colony morphology and serotyping resultant colonies. For the initial ten *S. pneumoniae* isolates tested, eight displayed morphological variants, including colonies that were phase bright, flat or very small. Variants were most commonly observed from swabs stored for 28 days at room temperature, and represented a small portion of total growth recovered. Some morphological variants were unable to be serotyped by both latex agglutination and Quellung, however most were able to be typed successfully (data not shown). Notably, no morphological variants were observed from swabs stored at 4^°^ C. These preliminary observations suggest some stress is placed on *S. pneumoniae* cells when stored in SDPs at room temperature, especially as storage length increases.

To confirm that the optimized conditions were applicable to a wide range of serotypes, we analyzed an additional 49 *S. pneumoniae* isolates, representing 40 different serogroups (Table S1). These isolates were stored on polyester swabs, inoculated with one-quarter lawns, then stored for 7, 14 and 28 days. Swabs were moistened with THB upon recovery as outlined above. To represent the most practical transport conditions, the experiment was conducted at room temperature only. Growth was recoverable from all swabs stored for 7 or 14 days, with satisfactory growth recovered for 48 of 49 (98%) and 44 of 49 (90%) isolates, respectively. Similarly to our initial findings, recovery decreased significantly at 28 days with only 20 of 49 (41%) isolates yielding satisfactory growth (*P* < 0.0001 when compared with day 7 and 14 results). No growth was recovered for isolates representing serotypes 14, 23A, 33F and 33C after 28 days of storage. Further investigations are needed to determine if survival is related to the particular isolates or clonal types tested, or a shared characteristic of all isolates from each serotype.

These optimized conditions were then tested against nine other clinically relevant bacterial species: *E. coli, H. influenzae, K. pneumoniae, N. gonorrhoeae, P. aeruginosa*, S*almonella spp., S. aureus*, *S.* agalactiae and *S. pyogenes* (Table S1). Clinical isolates were utilized, as it is likely they are more sensitive to adverse conditions compared with laboratory strains [8]. Duplicate polyester swabs were inoculated and stored in separate SDPs at room temperature for 7, 14 and 28 days, as described above. For all species, except *N. gonorrhoeae* and *H. influenzae*, full lawns (TNTC) were recovered at all time points. *H. influenzae* was viable after one day of storage only, while *N. gonorrhoeae* could not be recovered from SDP storage at any time point. Pilot studies indicated that higher inocula may increase storage limits for *H. influenzae*; this could be further investigated in future studies. All species could be identified correctly and retained original colony morphology upon recovery. The exception was *S. aureus*, which produced several colonies lacking zones of β-hemolysis upon recovery at days 7 and 28; although the reasons for this morphological change were not investigated.

SDP storage provides a technically simple and inexpensive method for transporting bacterial isolates. Our recovery rates for *S. pneumoniae* isolates were comparable to those reported for Dorset egg media after 14 days at room temperature, although viability was slightly lower in comparison when left for 28 days [15]. When stored at 4° C, SDP recovery exceeded that of the Dorset egg media [15]. SDPs can also increase transport durations at ambient temperatures to 14 days, compared with traditional Aimes’ or Stuarts’ media which require processing within 24 h [4]; or as an alternative to transporting STGG cultures at room temperature where pneumococci are non-culturable after 16 days [16], and the optimal time period may be as short as 28 h for some strains [17]. SDP transport does not require specialized equipment or maintenance of temperature during transport, the costs of which can be unattainable for low-income countries.

Variation in the initial inocula is a possible limitation to this study. However, portions of lawn culture were used as the inocula as a practical alternative to a defined CFU, given that our pilot data found viable counts from quarter lawns were comparable for the initial *S. pneumoniae* isolates tested (mean 2.43 x 10^8^ CFU (95% CI: 7.04 x 10^7^, 4.15 x 10^8^)). It is also possible that some saturation of swabs occurred during inoculation; our pilot data found viable counts from half and full lawns were 3.53 x 10^8^ CFU (95% CI: 1.03 x 10^8^, 6.03 x 10^8^ CFU) and 6.85 x 10^8^ CFU (95% CI: 1.17 x 10^8^, 1.25 x 10^9^) respectively. However, we do not anticipate this minor potential saturation of the swab impacted significantly on our findings. Using plate growth rather than broth also allows inoculation of larger numbers of bacterial cells. Some minor contamination was observed during the recovery process, however this did not affect the ability to serotype isolates or perform viable counts. Our study did not test the applicability of SDPs for transporting nasopharyngeal swabs or other specimens. Further investigations could be done to extend possible storage limits, investigate the impact of SDP storage on clonal types, and examine the effects of higher temperatures, which might be encountered during transport in warm climates.

## Conclusion

This study shows SDPs to be satisfactory for transport and short-term storage of *S. pneumoniae* and several other clinically relevant species. Our data demonstrate that *S. pneumoniae* remain suitably viable in SDPs for up to 14 days at room temperature, or 28 days at 4° C. Similarly, most of the other bacterial species tested remained viable after 28 days storage at room temperature. SDPs provide an inexpensive and simple method for the transportation of pure bacterial cultures.

## Supporting Information

Table S1 **List of bacterial isolates used to evaluate the use of Silica desiccant packets (SDPs) for bacterial transport.** For the provision of isolates we thank Fiona Russell and the Fiji Pneumococcal Project team; Samir Saha, staff of the Child Health Research Foundation, Dhaka Shishu Hospital, and families who participated in the Pneumococcal carriage project; and the Department of Microbiology, Laboratory Services, Royal Children’s Hospital, Melbourne. Thanks to the PneuCarriage project team, in particular Maha Habib for expert technical assistance, and Eleanor Neal for editorial assistance. We thank the Department of Microbiology, Laboratory Services, Royal Children’s Hospital for use of their VITEK^®^ machine and assistance with bacterial identification.
(DOC)
