# Supplementary material for: Silica Desiccant Packets for Storage and Transport of Streptococcus pneumoniae and Other Clinically Relevant Species
Source: PLoS One. 2013 Aug 7;8(8):e72353. doi: 10.1371/journal.pone.0072353 (PMC3737130; doi:10.1371/journal.pone.0072353)
Supplement: Table S1 — List of bacterial isolates used to evaluate the use of Silica desiccant packets (SDPs) for bacterial transport. For the provision of isolates we thank Fiona Russell and the Fiji Pneumococcal Project team; Samir Saha, staff of the Child Health Research Foundation, Dhaka Shishu Hospital, and families who participated in the Pneumococcal carriage project; and the Department of Microbiology, Laboratory Services, Royal Children’s Hospital, Melbourne. Thanks to the PneuCarriage project team, in particular Maha Habib for expert technical assistance, and Eleanor Neal for editorial assistance. We thank the Department of Microbiology, Laboratory Services, Royal Children’s Hospital for use of their VITEK® machine and assistance with bacterial identification. (DOC) [file pone.0072353.s001.doc]

Table S1. Isolates used to evaluate the use of SDPs for bacterial transport.

| **Species** | **Lab ID** | **Serotype** | **Site of Origin** | **Country of Origin** |
| --- | --- | --- | --- | --- |
| *S. pneumoniae* | PMP719 | 1 | Unknown | Unknown |
| *S. pneumoniae* | PMP37 | 2 | Nasopharynx | Fiji |
| *S. pneumoniae* | PMP278 | 3 | Nasopharynx | Fiji |
| *S. pneumoniae* | PMP722 | 4 | Unknown | Unknown |
| *S. pneumoniae* | PMP812 | 5 | Blood | Bangladesh |
| *S. pneumoniae* | PMP1121 | 6C | Nasopharynx | Bangladesh |
| *S. pneumoniae* | PMP1122 | 6D | Nasopharynx | Bangladesh |
| *S. pneumoniae* | PMP435 | 6A | Nasopharynx | Fiji |
| *S. pneumoniae* | PMP492 | 6B | Nasopharynx | Fiji |
| *S. pneumoniae* | PMP484 | 6B | Nasopharynx | Fiji |
| *S. pneumoniae* | PMP1123 | 7B | Nasopharynx | Bangladesh |
| *S. pneumoniae* | PMP727 | 7F | Unknown | Unknown |
| *S. pneumoniae* | PMP814 | 8 | CSF | Bangladesh |
| *S. pneumoniae* | PMP67 | 9N | Nasopharynx | Fiji |
| *S. pneumoniae* | PMP288 | 10A | Nasopharynx | Fiji |
| *S. pneumoniae* | PMP71 | 11A | Nasopharynx | Fiji |
| *S. pneumoniae* | PMP815 | 12F | Nasopharynx | Bangladesh |
| *S. pneumoniae* | PMP122 | 13 | Nasopharynx | Fiji |
| *S. pneumoniae* | PMP1114 | 14 | Nasopharynx | Bangladesh |
| *S. pneumoniae* | PMP816 | 15F | Blood | Bangladesh |
| *S. pneumoniae* | PMP73 | 15A | Nasopharynx | Fiji |
| *S. pneumoniae* | PMP80 | 16F | Nasopharynx | Fiji |
| *S. pneumoniae* | PMP81 | 17F | Nasopharynx | Fiji |
| *S. pneumoniae* | PMP61 | 18C | Nasopharynx | Fiji |
| *S. pneumoniae* | PMP1115 | 19B | Nasopharynx | Bangladesh |
| *S. pneumoniae* | PMP292 | 19A | Nasopharynx | Fiji |
| *S. pneumoniae* | PMP63 | 19F | Nasopharynx | Fiji |
| *S. pneumoniae* | PMP133 | 20 | Nasopharynx | Fiji |
| *S. pneumoniae* | PMP135 | 21 | Nasopharynx | Fiji |
| *S. pneumoniae* | PMP283 | 22F | Nasopharynx | Fiji |
| *S. pneumoniae* | PMP1116 | 23A | Nasopharynx | Bangladesh |
| *S. pneumoniae* | PMP110 | 23B | Nasopharynx | Fiji |
| *S. pneumoniae* | PMP713 | 23F | Nasopharynx | Fiji |
| *S. pneumoniae* | PMP778 | 24B | Unknown | Unknown |
| *S. pneumoniae* | PMP819 | 25F | Blood | Bangladesh |
| *S. pneumoniae* | PMP820 | 27 | Nasopharynx | Bangladesh |
| *S. pneumoniae* | PMP830 | 28F | Unknown | Unknown |
| *S. pneumoniae* | PMP284 | 29 | Nasopharynx | Fiji |
| *S. pneumoniae* | PMP821 | 31 | Nasopharynx | Bangladesh |
| *S. pneumoniae* | PMP831 | 32A | Unknown | Unknown |
| *S. pneumoniae* | PMP822 | 33C | Nasopharynx | Bangladesh |
| *S. pneumoniae* | PMP125 | 33F | Nasopharynx | Fiji |
| *S. pneumoniae* | PMP136 | 34 | Nasopharynx | Fiji |
| *S. pneumoniae* | PMP1117 | 35A | Nasopharynx | Bangladesh |
| *S. pneumoniae* | PMP1118 | 35F | Nasopharynx | Bangladesh |
| *S. pneumoniae* | PMP141 | 35B | Nasopharynx | Fiji |
| *S. pneumoniae* | PMP823 | 36 | Nasopharynx | Bangladesh |
| *S. pneumoniae* | PMP799 | 37 | Unknown | Unknown |
| *S. pneumoniae* | PMP140 | 38 | Nasopharynx | Fiji |
| *S. pneumoniae* | PMP1119 | 39 | Nasopharynx | Bangladesh |
| *S. pneumoniae* | PMP802 | 40 | Unknown | Unknown |
| *S. pneumoniae* | PMP1120 | 41A | Nasopharynx | Bangladesh |
| *S. pneumoniae* | PMP805 | 42 | Unknown | Unknown |
| *S. pneumoniae* | PMP806 | 43 | Unknown | Unknown |
| *S. pneumoniae* | PMP807 | 44 | Unknown | Unknown |
| *S. pneumoniae* | PMP127 | 45 | Nasopharynx | Fiji |
| *S. pneumoniae* | PMP809 | 46 | Unknown | Unknown |
| *S. pneumoniae* | PMP810 | 47A | Unknown | Unknown |
| *S. pneumoniae* | PMP834 | 48 | Unknown | Unknown |
| *Escherichia coli* | PMP987 | N/A | Blood | Australia |
| *Haemophilus influenzae* | PMP992 | N/A | Sputum | Australia |
| *Klebsiella pneumoniae* | PMP989 | N/A | Urine | Australia |
| *Neisseria gonorrhoeae* | PMP995 | N/A | Genital | Australia |
| *Pseudomonas aeruginosa* | PMP993 | N/A | Sputum | Australia |
| *S. agalactiae* | PMP994 | N/A | Tissue | Australia |
| *S. pyogenes* | PMP988 | N/A | Blood | Australia |
| *Salmonella spp.* | PMP991 | N/A | Feces | Australia |
| *Staphylococcus aureus* | PMP990 | N/A | Blood | Australia |

N/A = Not applicable
